# Measuring Indirect Radiation-Induced Perfusion Change in Fed Vasculature Using Dynamic Contrast CT

**DOI:** 10.3390/jpm12081254

**Published:** 2022-07-30

**Authors:** Antonia E. Wuschner, Mattison J. Flakus, Eric M. Wallat, Joseph M. Reinhardt, Dhanansayan Shanmuganayagam, Gary E. Christensen, John E. Bayouth

**Affiliations:** 1 University of Wisconsin, Madison, WI 53706, USA; flakus@wisc.edu (M.J.F.); wallat@wisc.edu (E.M.W.); dshanmug@wisc.edu (D.S.); jebayouth@gmail.com (J.E.B.); 2 University of Iowa, Iowa City, IA 52242, USA; joe-reinhardt@uiowa.edu (J.M.R.); gary-christensen@uiowa.edu (G.E.C.)

**Keywords:** lung SBRT, perfusion, post-RT toxicity, swine model, functional avoidance

## Abstract

Recent functional lung imaging studies have presented evidence of an “indirect effect” on perfusion damage, where regions that are unirradiated or lowly irradiated but that are supplied by highly irradiated regions observe perfusion damage post-radiation therapy (RT). The purpose of this work was to investigate this effect using a contrast-enhanced dynamic CT protocol to measure perfusion change in five novel swine subjects. A cohort of five Wisconsin Miniature Swine (WMS) were given a research course of 60 Gy in five fractions delivered locally to a vessel in the lung using an Accuray Radixact tomotherapy system with Synchrony motion tracking to increase delivery accuracy. Imaging was performed prior to delivering RT and 3 months post-RT to yield a 28–36 frame image series showing contrast flowing in and out of the vasculature. Using MIM software, contours were placed in six vessels on each animal to yield a contrast flow curve for each vessel. The contours were placed as follows: one at the point of max dose, one low-irradiated (5–20 Gy) branching from the max dose vessel, one low-irradiated (5–20 Gy) not branching from the max dose vessel, one unirradiated (<5 Gy) branching from the max dose vessel, one unirradiated (<5 Gy) not branching from the max dose vessel, and one in the contralateral lung. Seven measurements (baseline-to-baseline time and difference, slope up and down, max rise and value, and area under the curve) were acquired for each vessel’s contrast flow curve in each subject. Paired Student *t*-tests showed statistically significant (*p* < 0.05) reductions in the area under the curve in the max dose, and both fed contours indicating an overall reduction in contrast in these regions. Additionally, there were statistically significant reductions observed when comparing pre- and post-RT in slope up and down in the max dose, low-dose fed, and no-dose fed contours but not the low-dose not-fed, no-dose not-fed, or contralateral contours. These findings suggest an indirect damage effect where irradiation of the vasculature causes a reduction in perfusion in irradiated regions as well as regions fed by the irradiated vasculature.

## 1. Introduction

Lung cancer is one of the most commonly diagnosed cancers and is currently responsible for the highest percentage of cancer-related deaths. In 2022, 236,760 new cases of lung cancer and 130,180 deaths are projected (21.6% of total cancer-related deaths and the most deaths of any individual cancer) [1]. Of those diagnosed, between 37 and 57% of patients will receive radiation therapy (RT) as part of their treatment depending on the stage of their cancer [1].

While external beam RT is an effective non-surgical therapy, there are risks of patients developing radiation-induced lung injuries (RILI). Approximately 33.5% of patients will develop RILI [2], which can cause significant respiratory symptoms in lung cancer patients with compromised baseline lung function. These toxicities can be introduced by damaging the lung pleura, vasculature, and airways. Exact mechanisms that cause this damage are currently not well understood, but two common resulting RILI that can develop are pneumonitis and fibrosis; these RILI can significantly decrease patient quality of life and can even be fatal [3].

One potential strategy to avoid RILI is to use functional avoidance RT treatment planning. The goal of functional avoidance radiation therapy is to mitigate radiation-induced normal tissue toxicities by selectively avoiding high-functioning regions of the lung. In current clinical trials, functional avoidance was achieved while maintaining adequate tumor coverage and satisfying clinical dose constraints to major organs [4,5,6,7]. In recent years, multiple studies have looked at using functional metrics to create risk assessments and have found that the inclusion of these metrics increased the predictive power of the toxicity predictions and radiation response output from these models [5,8,9,10,11,12,13,14].

One challenge with performing functional avoidance is creating comprehensive predictive models for RILI. Previous work investigated the radiation dose-response of pulmonary function and have developed predictive models assessing local lung function [4,6,15,16,17,18,19,20,21]. Lung function has been assessed using imaging modalities such as SPECT, which can provide both ventilation and perfusion information, or through the use of aerosols in CT, PET, and MRI [22]. However, for these models to become integrated into clinical practice, they must be executable in current clinical workflow. For clinical integration, CT is an exceptional option due to its high spatial resolution and routine use in treatment planning. Some groups have begun developing predictive models derived from CT, but to date, all functional-avoidance studies using CT have primarily focused on assessing only ventilation, neglecting perfusion and therefore providing an incomplete assessment of lung damage [7,18,23].

Our previous work quantified radiation-induced dose–response, correlating 4DCT-derived Hounsfield Unit (HU) changes with changes in contrast from dynamic contrast-enhanced CT [24]. However, this work focused on direct radiation damage without quantifying observations of indirect damage to regions supplied by damaged vasculature due to irradiation being in the base of the lung. Recent functional lung imaging work has presented evidence of there being an “indirect effect” in which regions that are unirradiated or irradiated with low dose (below dose thresholds that are known to cause damage) and supplied by highly irradiated vasculature are damaged post-RT [25,26]. However, this evidence was not commented on in those works. Additionally, this work was done using SPECT, which has the issue of producing low-resolution images prone to artifacts and attenuation, making the nuances of the indirect effect difficult to localize [23,25,26,27]. Wallat et al. presented the first CT-derived evidence of there being an indirect effect, focusing on post-RT ventilation change due to damage to the airways [28]. Vicente et al. later developed a functional avoidance technique that incorporated this indirect ventilation effect and found that the average predicted ventilation preservation was 14.5% higher than conventional RT techniques and 11.5% higher than functional avoidance algorithms that consider only local damage [29]. These results suggest that the indirect effect is crucial to consider, but is all based on ventilation models only. As described in Ireland et al., perfusion change is also an important functional metric that has been shown to be more predictive of patient functional decline and outcome [30]. Therefore, analysis of the indirect perfusion change is needed, particularly utilizing a method that has adequate resolution to identify the source of the damage.

We hypothesize functional avoidance studies using 4DCT can model both ventilation and perfusion metrics to provide a comprehensive assessment of lung damage. In this study, we explore CT-based perfusion changes following RT, and how those changes extend beyond directly irradiated lung tissue. By establishing the first perfusion-based radiation response model incorporating indirect effects, post-RT predictive power can be improved to create superior functional avoidance treatment plans, leading to improvements in preserved function and patient outcomes.

## 2. Materials and Methods

### 2.1. Novel Swine Model

Swine are well suited for biomedical studies pertaining to the development/validation of diagnostic and therapeutic technologies. The genetic proximity of swine to humans combined with their overwhelming anatomical, physiological, and pathophysiological similarities make swine the ideal model for preclinical studies of novel technologies [31,32,33,34]. Additionally, swine experience expedited growth compared to humans. This feature can be leveraged in biomedical research because it results in expedited development of disease, healing, and toxicity, allowing for expedited data acquisition and development of novel technologies [33,34].

Historically, most studies looking at radiation response in the lung have used conventional swine breeds [33,35]. Due to rapid growth in adulthood, swine can reach from 550 to over 650 pounds, which makes them difficult for CT imaging. To combat this challenge, many studies use young swine in order to execute the experiments of interest. However, these swine mimic a human child and experience rapid rates of healing, development, and cell regeneration, which is not an accurate model of a typical patient with lung cancer we would treat clinically [35]. This work used a genetically modified swine breed that poses numerous benefits.

The Wisconsin Miniature Swine (WMS) possess several characteristics that make them an ideal model. WMS were created by selective crossbreeding of several swine breeds such that their weight, size, and physiology are similar to humans and their body composition can be easily manipulated [35]. As they can be easily maintained at human size for any length of time, they will remain the same size from intervention to necropsy. In addition, we were able to select swine that had lung volumes that were within the range of typical human subjects for this work.

### 2.2. Swine Setup

Five WMS (14.4 +/− 1.7 months old) were analyzed. The WMS were sedated to eliminate motion artifacts and mechanically ventilated to a consistent tidal volume of 1 L and respiratory rate of 15 breaths per minute, matching the average tidal volume and respiratory rate of human subjects. The swine were imaged prior to treatment and 3 months following treatment. After the 3 month post-RT imaging session, the swine were euthanized and the lungs were extracted for future histopathology analysis. All details regarding animal care and drugs administered can be found in the Supplementary Materials. The animal care practices and all experimental procedures were approved by the University of Wisconsin Institutional Animal Care and Use Committee (IACUC). The drugs and methods of anesthesia and euthanasia were approved in compliance with American Veterinary Medical Association (AVMA) guidelines for anesthesia and euthanasia of swine. Both committees assured that all procedures were in compliance with the Animal Research: Reporting of In Vivo Experiments ARRIVE guidelines.

### 2.3. Swine Treatment

**Figure 1:**
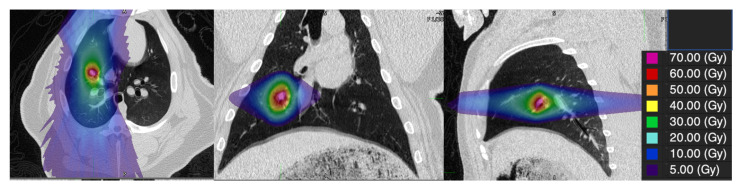
Example dose distribution delivered to the Swine subjects.

Each WMS received a research course of 60 Gy to 95% of the planning target volume (PTV) in five fractions approved by IACUC. The PTV was centered on a vessel and airway in the right upper lobe of the subject, and the left lung was left unirradiated (max point dose < 5 Gy). The choice of PTV location was based on finding a location that allowed us to irradiate on a vessel/airway pair as cranial as possible, but while keeping max point dose in the contralateral lung below 5 Gy as well as limiting the dose to the heart to less than 1 mL receiving 10 Gy. The choice to irradiate to 60 Gy was to match the typical prescription of a human lung SBRT patient that is prescribed in our clinic. Figure 1 shows a representative dose distribution that was delivered to the subjects. Treatments were delivered on the Radixact^®^ linear accelerator with motion Synchrony treatment system (Accuray Incorporated, Sunnyvale, CA, USA) in order to maximize dose conformity and reduce the uncertainty of dose delivery due to respiratory motion.

Radixact^®^ is a helical tomotherapy radiation therapy delivery system capable of delivering conformal intensity-modulated radiation therapy (IMRT). It contains an intrafraction motion management system called Synchrony^®^, which has been adapted from CK Synchrony [36]. On the system, an X-ray tube and flat-panel kV imager are offset 90° from the megavoltage (MV) imager and beam. The kV imaging subsystem is used to periodically localize the target during treatment. For monitoring respiratory motion, light-emitting diodes (LEDs) were placed on the swines’ chest and identified with a camera mounted to the treatment table to provide the phase of respiration. The target can then be localized without implanted fiducials near the target using a motion correlation model. Further details of the model are described in Schnarr et al. [36]

Treatment fractions were delivered following a standard clinical SBRT schedule receiving each fraction with a day in between each delivery during weekdays and 2 days over the weekend. Subjects were mechanically ventilated to 15 breaths per minute during treatments.

### 2.4. Dynamic Contrast CT

All CT images were acquired on a Siemens SOMATOM Definition Edge CT scanner. Each swine underwent two imaging sessions (one session before receiving radiation and one 3 months post-RT). In each session, a contrast-enhanced dynamic 4DCT image was obtained.

**Figure 2:**
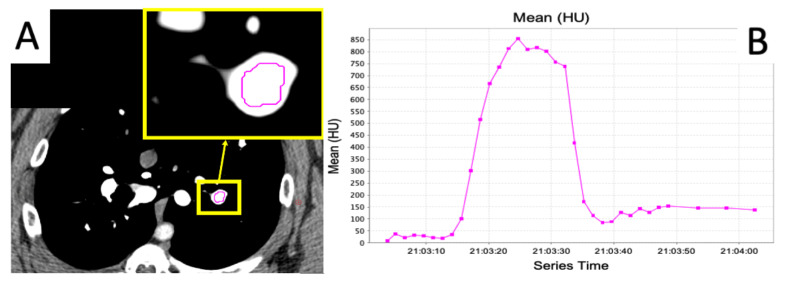
Example of a curve showing the flow of contrast into and out of a vessel. (**A**) Example slice in a frame of the scan showing the placement of an ROI in a vessel. (**B**) Average Hounsfield Unit (HU) value inside the ROI over the frames of the scan. Increasing HU represents contrast flow into the vessel, and decreasing HU represents contrast flow out of the vessel.

The dynamic 4DCT images were acquired over the central 15 cm of the lung as 80 mL of iodine contrast (Omnipaque 300) was injected at a rate of 5 mL/s. The acquisition consisted of repeated scanning of the same volume at 1.5 s intervals until the contrast had washed out of the lung. In total, the dynamic 4DCT images contain between 28 and 36 frames. Acquisition began before contrast was injected to collect baseline images. After the acquisition of baseline, contrast injection began and acquisitions continued until the contrast had washed out of the lung vasculature. We believe this scanning protocol is a better indication of perfusion than the standard blood volume dual energy scan because it scans the same volume over a period of time as contrast flows in and out of the vasculature as opposed to capturing a snapshot at one time point of where the contrast is in the lung. An example of wash-in/wash-out kinetics captured by these scans is shown in Figure 2.

### 2.5. Regional Perfusion Analysis

**Figure 3:**
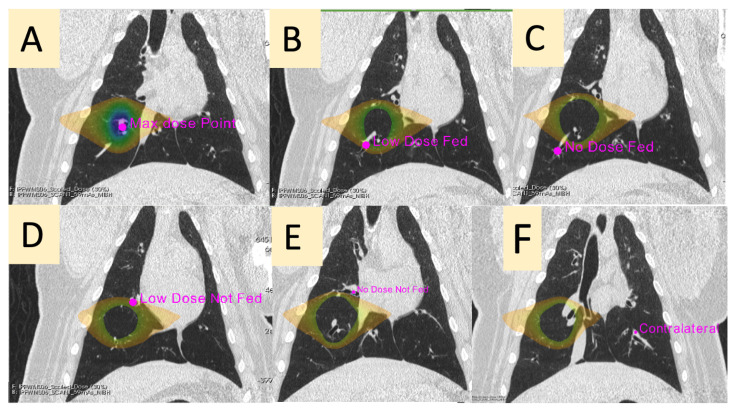
Representative placement of contours analyzed. The point of max dose is shown in the center of the full dose distribution (**A**). The 5–20 Gy dose distribution is shown in (**B**–**D**) to indicate the region of low dose. A point on the low-dose region fed by the max dose vessel (**B**) and not-fed (**D**) are placed. Points receiving no-dose fed by the max dose vessel and not-fed are placed in (**C**,**E**), respectively. Finally, a point in the contralateral lung is placed approximately where the max dose is mirrored on the ipsilateral lung (**F**).

**Figure 4:**
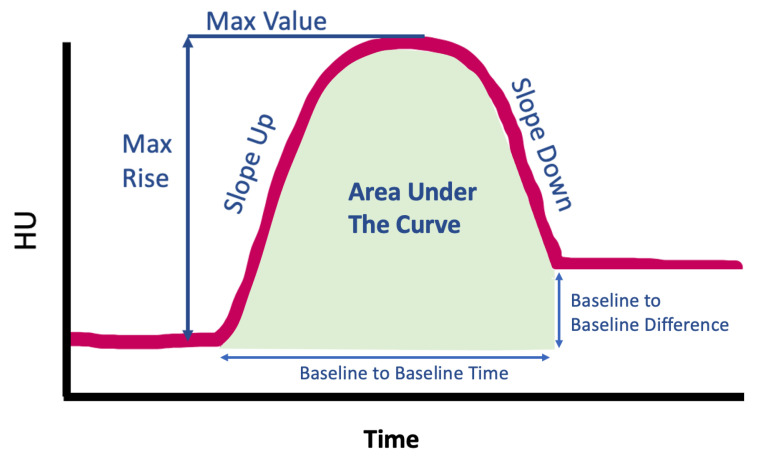
Diagram showing the different measurements that were obtained in each contour.

Pre- and post-RT dynamic perfusion CTs were deformably registered to each other for all subjects using a B-spline deformable image registration algorithm to allow for longitudinal voxel-wise comparisons [37,38]. For each swine, 6 contours were analyzed with 6 measurements obtained using the pre- and post-RT contrast curves. Contours were placed using the pre-RT dynamic perfusion CT, using the dose distribution in MIM Software (Cleveland, OH). Contours were then deformably propagated to each frame of the dynamic perfusion CT in MIM and transferred to the previously registered post-RT scan. Figure 3 and Table 1 show and describe the locations of the measurements, and Figure 4 shows the measurements that were taken at each point. There was one swine for which the “No-Dose Fed” contour could not be analyzed due to a mistake in image acquisition. This was due to incorrect selection of the appropriate field of view for the dynamic contrast scan, and thus there was not enough overlapping anatomy imaged pre- and post-RT to create a contour meeting that classification. All other contours were able to be created on this subject. Additionally, there was one swine for which the low-dose fed, no-dose fed, low-dose not-fed, and no-dose not-fed contours all showed no contrast flow post-RT. The max dose and contralateral contours as well as other vasculature in the ipsilateral lung did, so we do not believe it was an error in acquisition. This subject is further discussed in the discussion, but due to this effect, the baseline-to-baseline time value could not be calculated for this subject as there was no clear starting or ending baseline. The other 4 swine were able to have all contours analyzed. The percent change pre- to post-RT was then calculated using Equation (1). Student paired two-tailed *t*-tests were used to compare the pre- and post-RT values of each measurement across the 5 swine.

(1) $$\%\mathrm{change}=\frac{{\mathrm{Value}}_{\mathrm{post}}-{\mathrm{Value}}_{\mathrm{pre}}}{{\mathrm{Value}}_{\mathrm{pre}}}\times 100\%$$

#### 2.5.1. Calculation of Slopes

As seen in Figure 2B, the contrast curves exhibit a region where the contrast is flowing into the vasculature and a region where the contrast is flowing out. Each of these regions has a section that can be approximated as linear. Within these regions, the slopes were calculated using the change in HU from the start of the linear region to the end of the linear region as well as the time that passed during these acquisitions (recall acquisitions are acquired at fixed time frequency, so it can be derived from how many acquisitions pass in between these points). Thus, the slope can be calculated as

(2) $$\mathrm{Slope}=\frac{\Delta \mathrm{HU}}{\Delta \mathrm{time}}\mathrm{HU}/\sec\mathrm{ond}$$

#### 2.5.2. Area under the Curve

The area under each contrast curve was calculated using the built-in trapz function in MATLAB 2020a (Mathworks, Natick, MA, USA). This function performs numerical integration via the trapezoidal method. This method approximates the integration over an interval by breaking the area down into trapezoids with more easily computable areas [39].

## 3. Results

### Imaging Results

**Figure 5:**
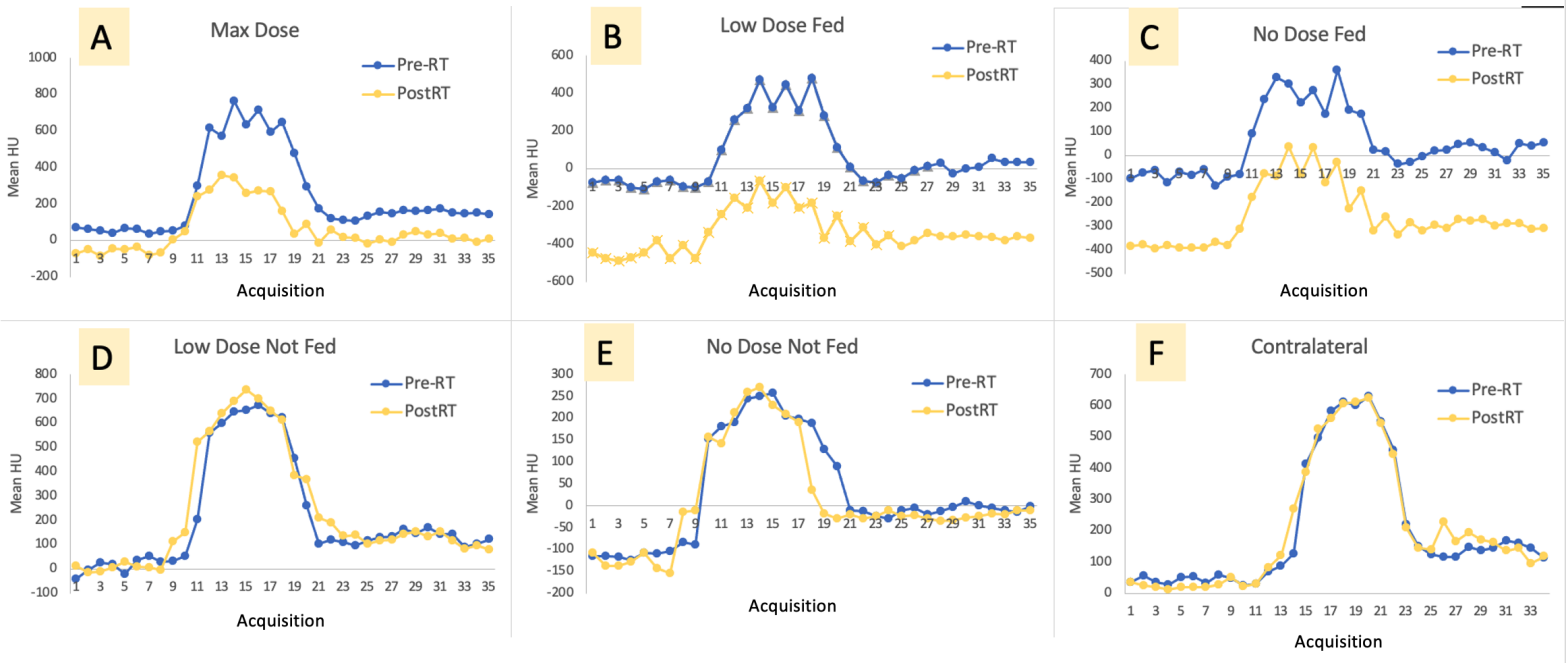
Representative contrast curves pre- and post-RT in an example subject in the max dose contour (**A**), low-dose fed contour (**B**), no-dose fed contour (**C**), low-dose not-fed contour (**D**), no-dose not-fed contour (**E**), and contralateral lung (**F**). There is little change in the not-fed contours and contralateral lung contour, while the fed and max dose contours observe significant change.

Figure 5 shows the pre- and post-RT contrast curves for each of the six vessel locations of interest. This is a representative subject that highlights the differences seen in the curves post-RT.

**Figure 6:**
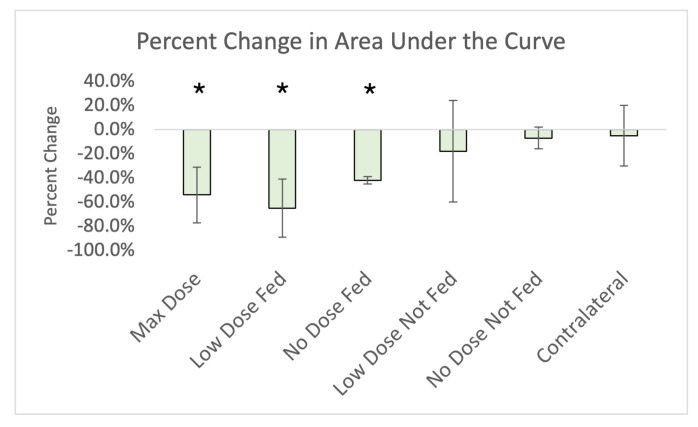
Percent change in area under the curve for each contour analyzed. Each bar represents the average of the 5 subjects (or 4 in the case of the no-dose fed contour). Error bars are the standard deviation of the percent changes in subjects. Statistically significant results are denoted with a *.

Figure 6 shows the percent change in area under the curve for all analyzed contours. Statistically significant reductions (p<0.05) were observed in all of the fed contours as well as the max dose contour.

The percent changes in each of the measurements shown in Figure 4 are summarized in Table 2. The average percent change and standard deviation values are the average and standard deviation of the percent changes in each of the five (or four in the case of the no-dose fed contour) swine. Each entry is in the form “Average (Standard Deviation)”, where statistically significant values are denoted by a * and are in red.

## 4. Discussion

### 4.1. Changes in Kinetics

#### 4.1.1. Ipsilateral Contours

The main result of this work is highlighted in Figure 6. Statistically significant (*p* < 0.05) reductions in the area under the curve were observed in the max dose contour as well as the fed contours. These reductions in area under the curve represent an overall reduction in contrast observed in the vessel and a change in the numerator from Equation (2). This response to radiation has been reported previously in irradiated vasculature [24], where it was observed that in vasculature irradiated above 25 Gy, there appeared to be a leakage of contrast from the vasculature into the non-vessel parenchyma. The observation of reduced post-RT area under the curve in fed contours but not in not-fed contours suggests an indirect damage effect to the low or unirradiated tissues where there is leakage in the highly irradiated vessel preventing contrast (and more importantly blood) from reaching the branching vessels.

Statistically significant (*p* < 0.05) reductions in all seven metrics were observed in the max dose contours. In the low-dose fed contours, there were statistically significant reductions in max rise, slope up, and slope down. The no-dose fed contours also saw statistically significant reductions in slope up, slope down and baseline-to-baseline difference. No contours other than the max dose showed statistically significant changes in baseline-to-baseline time or max value. The reductions in slope up (into the vasculature) and down (out of the vasculature) with no changes in baseline-to-baseline time suggest that the contrast is flowing in and out of the vessel more slowly, further suggesting that the contrast leaked out of the vessel prior to it being able to reach these vessels.

The low-dose not-fed and no-dose not-fed contours did not see a statistically significant reduction in any slope metric, nor in the max rise, max value, area, or baseline-to-baseline time. The no-dose not-fed contour did see a statistically significant reduction in the baseline-to-baseline difference metric. However, we attribute this to a skewed reading from one of the subjects that had particularly small vasculature available for this contour to be placed in and likely suffered from partial volume averaging (explained further in Section 4.3).

**Figure 7:**
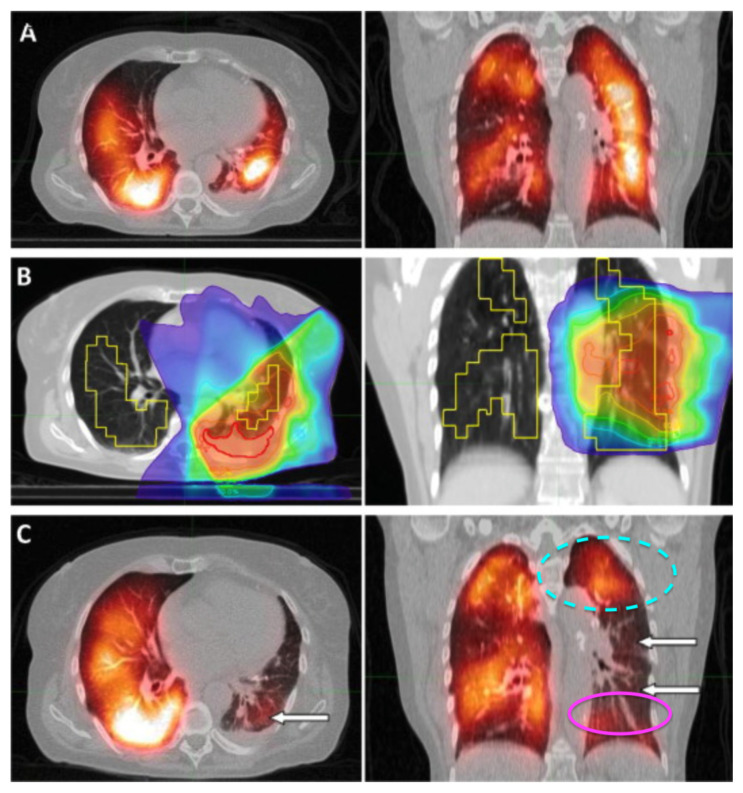
Taken from Farr et al. [25]. SPECT/CT of a patient with tumor in the right lung before radiotherapy (**A**), planning CT with dose to the gross tumor volume in color wash, SPECT defined functional lung outlined in yellow (**B**), SPECT/CT 3-months post-RT (**C**). A dotted cyan oval is drawn to indicate a region that received low dose but did not experience perfusion decline. A magenta oval is drawn in another region that received low dose but did experience perfusion decline post-RT and is fed by an irradiated region. Reprinted with permission.

**Figure 8:**
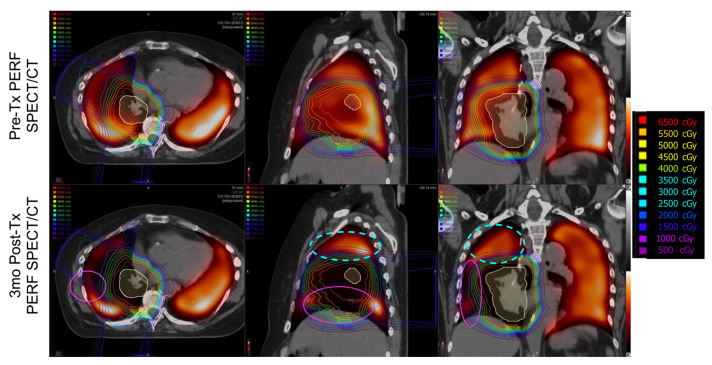
Taken from Thomas et al. [26]. Observed radiation dose–response on longitudinal perfusion SPECT/CT. (**Upper row**) Pre-treatment lung perfusion SPECT co-registered to planning CT and radiation isodose line rainbow overlay. (**Lower row**) Three-month post-treatment perfusion SPECT co-registered to planning CT and radiation isodose lines (rainbow overlay). SPECT window/level were normalized to out-of-field integral uptake. Regions within the treatment field show reductions in uptake that are correlated with radiation dose magnitude and spatial distribution. A dotted cyan oval is drawn to indicate a region that received low dose but did not experience perfusion decline. A magenta oval is drawn in another region that received low dose but did experience perfusion decline post-RT and is fed by an irradiated region. Reprinted with permission.

Without this flow of oxygenated blood, we can infer that perfusion is reduced in these regions as well, since the capillary network branches off these vessels. While this is the first study to our knowledge to report this indirect damage, it has been observed previously. Both Farr et al. and Thomas et al.’s work with SPECT-CT images of the lung post-RT also experienced this effect. While not commented on directly in their work, it is reported in their results as shown in Figure 7 and Figure 8 [25,26]. In both figures, it can be observed that the heat maps of SPECT perfusion show reductions post-RT in lowly irradiated areas inferior/supplied by the spot of maximum dose. However, these reductions are not present or are significantly less severe in regions lowly irradiated but superior/not supplied by the maximally dosed region. Furthermore, there are no significant reductions observed in the contralateral lungs. This work was done with SPECT, so the resolution is poor and it is impossible to isolate the feeding vasculature relationship as we are able to do with our methods. However, it does show evidence that there is an indirect effect matching the effect described in this work which was derived via dynamic contrast-CT. SPECT is currently considered the gold standard for perfusion imaging, so this confirmation of observations further support that our CT-derived method is a potential adequate surrogate for perfusion measures.

#### 4.1.2. Contralateral Lung

No metric saw statistically significant changes in the contralateral lung contours. Changes in max rise, max value, baseline-to-baseline time, and difference in baselines were all below 6%. The average changes in the slopes had larger magnitudes but were not statistically significant and were skewed by one subject who had a shorter baseline-to-baseline time, which thus caused the slope to increase (change in the time variable from Equation (2)).

### 4.2. Use of a Novel Swine Model

The novelty of the swine model use in this work allows for more direct translation into human studies than previous animal models. Previous work has already established a strong correlation between the WMS and human radiation-induced lung density changes [24]. The WMS at the size used in this work had lungs that matched human adult lungs. Additionally, the WMS at the size used (matching human adult lungs) were swine in their early adulthood (14.4 +/− 1.7 months old). Swine reach sexual maturity at approximately 5 months of age, so a 14-month-old WMS is close to a human in their late twenties, maybe very early 30s. Using a conventional breed of swine as previous studies have done would have resulted in the swine being approximately 3 months of age (in order to match the size of human lungs). Given that swine reach sexual maturity at 5 months of age, a 3-month-old swine is equivalent to a pre-pubescent human child at 6–8 years of age. A conventional swine at this age has a rate of development where tissue remodeling and size changes are very rapid. The swine’s ability to heal and response to radiation damage (i.e., pathophysiology) would not mimic that of a human adult. The WMS allowed us to more closely model the pathophysiology observed in a human adult. A 14-month-old WMS is close to a human in their mid-late twenties, which more accurately mimics a patient we would expect to treat.

Additionally, using swine was ideal due to the ability to provide a more controlled experiment than would be possible in humans. It has been established previously that regional perfusion can vary with tidal volume [40]. Having the ability to fix the tidal volume of the swine’s lungs at breath hold allowed for all image acquisitions and measurements to be consistent and helped minimize confounding variables in the measurements and isolate the change in perfusion.

### 4.3. Benefits of Dynamic Perfusion CT

The current “gold standard” of perfusion imaging is SPECT, such as the work done and displayed in Figure 7 and Figure 8 from Farr et al. and Thooas et al., respectively [25,26]. However, SPECT imaging struggles with spatial and temporal resolution due to patient respiratory motion as well as the intrinsic resolution of the detectors. Performing measurements using CT and at breath hold as done in these subjects helps mitigate these concerns.

**Figure 9:**
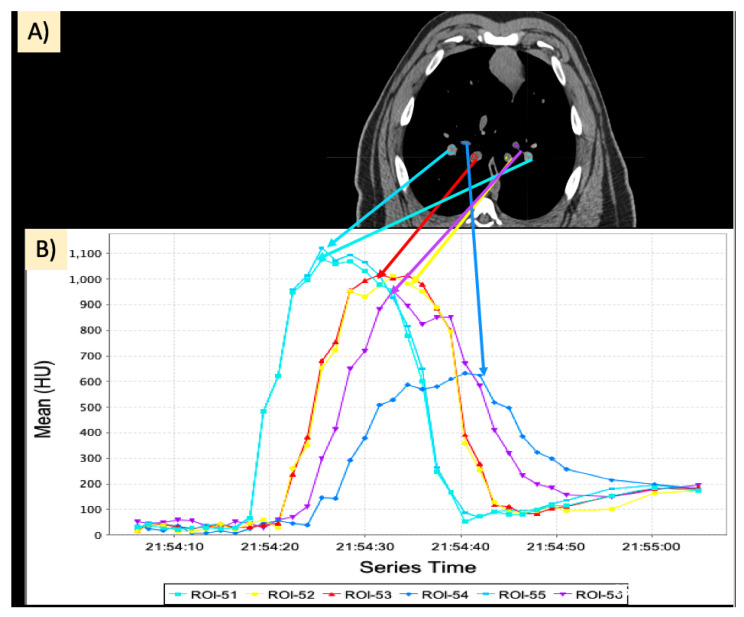
Dynamic contrast-CT data in different vasculature. (**A**) Different contours placed in different vasculature in the lung. (**B**) Corresponding contrast flow curves.

Previously reported CT-derived perfusion data have used pulmonary blood volume (PBV) scans. The novel contrast-enhanced CT protocol used in this work presents an added benefit of full kinetic analysis that PBV scans do not provide as well as an improvement in the amount of vasculature captured. A PBV scan only provides a snapshot in time at what is believed to be the time where the contrast concentration in the vasculature is the highest. Different vasculature, however, reach this maximum concentration of contrast at different points in time due to the time delay that occurs for contrast to flow into the smaller vessels. This effect is illustrated in Figure 9 below. Notice the different colored contours placed in different vessels of the CT and the corresponding curves in the plot on the right. It is clear that no one timepoint adequately captures all vasculature. However, with this method, we perform repeated scanning and thus can analyze the kinetics of each vessel individually, providing a more comprehensive assessment of the lung.

### 4.4. Limitations of the Study

There are a few features that contributed toward some of the larger variations in measurements in some of the metrics analyzed and are limitations of the study conducted. In addition to the features discussed below, a main limitation of this study is that the sample size is small. While results are convincing, this should be repeated in a larger population to reduce variation due to single-subject variability.

#### 4.4.1. Partial Volume Effect and Registration Error

Naturally, the vessels that branched from the primary vessels become smaller in diameter as they continue to branch. For all subjects, the low and no-dose vasculature (both fed and not-fed) were much smaller than the max dose vessel. This caused the contour created in this vessel to be smaller and thus less voxels to be averaged over in calculating the average HU. We did our best to place contours only in the lumen of the vessel, but during registration of these contours between frames as well as between time points, there is the possibility that there was some partial volume effect taking place where part of the vessel wall was contained in the contour, thus lowering the average HU of the contour and affecting the accuracy of some of the HU-based measurements (max value, max rise, slopes, and baseline-to-baseline difference). However, since for a given subject the pre- and post-RT anatomy was the same vessel chosen, the area under the curve metric should be robust to this effect, and since the area under the curve results echoed those sensitive to this effect, we still have confidence that the overall trends seen in the animals are insensitive to this noise.

This effect can be seen moderately in Figure 5, where it can be observed that some curves are not smooth but rather more jagged. The partial volume effect particularly manifested in one subject where the second baseline value was recorded as lower than the initial baseline value in the no-dose fed contour. Physiologically, this cannot be explained as the original baseline values were taken prior to any contrast injection, and the injection would not cause a reduction in HU. Additionally, this was observed in the pre-RT scan in only one contour, so it cannot be attributed to any radiation effect. For this subject, we attribute this observation to partial volume effect and registration error of the small vasculature between frames of the dynamic 4DCT.

#### 4.4.2. No Flow of Contrast in One Swine

One swine did not show any contrast flow during the post-RT scan in the low-dose fed and no-dose fed contours (they did show contrast flow through the max dose contour as well as contralateral contour). Thus, the HU values of the “curve” remained very close to baseline. This caused the max rise, area, and baseline-to-baseline difference values for that subject to be near 0 and the baseline-to-baseline time to be not computable, which contributed to larger variation in those measurements (especially no-dose fed, which had a lower sample size of 4 instead of 5). In that subject, the max dose contour’s metrics also showed larger reductions than the other subjects (66% reduction in the max rise compared to the 41% average and 81% reduction in area compared to the 56% average). These results suggest that this subject saw a more severe response where more contrast leaked out of the highly irradiated vessels than the others, causing no measurable contrast to reach these branches. It is also worth noting that the contralateral lung’s percent change in area under the curve for this subject was positive (38%), suggesting a compensatory effect in this subject.

If we were to exclude this subject from analysis, some of the values in Table 2 would change in the contours that saw no flow. The area under the curve metric, our comprehensive metric, would change in the low-dose fed contour (−65 +/− 24% to −58 +/− 23%), no-dose fed contour (−55 +/− 27% to −42 +/− 3%), low-dose not-fed contour (−36 +/− 54% to −18 +/− 42%), and no-dose not-fed contour (−24 +/− 39% to −7 +/− 9%). Even with the exclusion of this subject, only the fed contours show statistically significant changes. It is unclear as to exactly why this particular swine saw this response where the others did not, and it would be useful to conduct further studies with more subjects to determine if other subjects exhibit this response, and if so, what correlations between those subjects can be seen to predict this severe response.

#### 4.4.3. Comment on “Not-Fed” Contours

The reductions reported in Table 2 show that, while not statistically significant, the average percent reductions in area under the curve in the not-fed contours were not 0. These contours also had a wide variability in results. In addition to the partial volume effects being more prominent in these vessels’ contours, we believe a variable inflammatory effect is being observed that is affecting these vessels. Multiple studies have shown that radiation can induce an inflammatory effect, but the severity, onset, and time span of this effect is variable by patient [3]. Previous work has also shown that the 3-month timepoint in the swine is equivalent to somewhere between the 6- and 12-month response seen in a human [24]. This timepoint is known to have transient effects present where inflammation may be still resolving in some subjects, yet fully resolved in others. With inflammation, shifting of the vasculature can occur in the lung parenchyma, including some deformation and constriction of the smaller vasculature. Therefore, if a given subject experienced a more severe inflammatory effect, the vessels may experience a reduction in blood flow until the inflammation subsides. However, since this is variable by subject, some will show no change to this vasculature since the vessel itself was not damaged.

#### 4.4.4. Overestimation of Perfusion Reductions

Due to features of the irradiation scheme chosen in this work, it is possible that the values reported in this study overestimate the changes in perfusion that would be experienced in a typical patient. The first feature of note is the fractionation scheme. In this work, 5×12 Gy was chosen, but there are other prescriptions that human lung SBRT patients could receive that may yield differing results due to the radio-biological changes that would occur from a change in fractionation. The second feature is the fact that we irradiated directly on a vessel. In general, vessels are not targeted since it is the central lesion that is being treated. However, in many cases, vessels do end up receiving high doses in the resulting dose distributions. Additionally, current dose toxicity reports such as RTOG 0813 only report dose constraints and toxicity results for the great vessels [41], so vasculatures of the size irradiated in our work are currently not standard practice to consider when developing treatment plans. However, this work clearly shows there is a consequence to irradiating these vessels, particularly if those vessels feed other regions of the lung. The purpose of this work was to characterize the penalty of irradiating these smaller vasculatures that feed other large regions as well as those that do not in order to understand the consequences of each. This information could potentially aid decisions in which vasculature get irradiated if it is possible to manipulate. Therefore, while our results may be over-estimations depending on the exact clinical scenario, they provide an upper bound for the potential damage that could be caused.

### 4.5. Clinical Impact

This work quantifies an anatomical response to radiation dose in an animal model that has been previously established as a surrogate for human response [24]. Additionally, we used a novel contrast-enhanced CT protocol that allowed for a full kinetic analysis of each vessel. This is an improvement on previous conventional pulmonary blood volume techniques, as those techniques only provide a snapshot in time, and different vasculatures will reach their maximum concentration of contrast at different points in time due to the time delay that occurs for contrast to flow into the smaller vessels. Other groups have demonstrated radiation-induced changes in perfusion using SPECT [16], and their results have suggested an indirect effect of low-irradiated or unirradiated vasculature fed by highly irradiated vasculature experiencing a reduction in perfusion [25,26]. However, these studies did not comment on this effect and were not measured with a method that had the spatial and temporal resolution to quantify or pinpoint the cause. Our work uses a novel CT-based technique that can isolate the reduction to the vasculature involved. Knowledge of this response and the damage that is caused to the fed regions of the lung could help to aid treatment planning decisions to avoid major vasculature. Previous work using this animal model connected the changes observed in contrast to metrics derived on non-contrast 4DCT [24]. The ability to infer these changes from 4DCT would immensely aid translation to a clinical setting since 4DCTs are already routinely collected for treatment planning and would not require the acquisition of additional scans. These results would also present an opportunity for predictive models to be built to predict the functional cost of irradiating major vessels and allow for superior functional avoidance therapy.

After the 3-month post-RT scan, the swine lungs were extracted from the animal for future pathology studies. This work will provide further insight regarding the physiological response of these subjects and the damage done to the vasculature in each of the contours analyzed. Future work will also include a clinical trial analyzing the response of a larger sample size of these novel swine as well as an analysis of contrast-enhanced scanning on humans. This will enable faster development of predictive models that would be able to be validated on existing human subject data from this trial. From there, clinical trials assessing the effectiveness of intervention mechanisms on human subjects may be initiated.

## 5. Conclusions

It has been previously established that radiation induces changes in pulmonary anatomy post-RT. However, it has not been fully established what the indirect effect to anatomy fed by highly irradiated regions is. This work measured a reduction in perfusion in irradiated vascular regions as well as regions fed by the irradiated vasculature in five WMS. All work was done using a WMS model that has previously been established as a surrogate for analyzing radiation-induced changes in humans treated with SBRT. These measurements combined with previous work present a potential bio-marker for analyzing functional changes in perfusion that can be derived from 4DCT as opposed to requiring additional scans outside of clinical protocol. This would allow these metrics to be considered in functional avoidance therapy and could provide a significant benefit to patient outcome.

## Figures and Tables

**Table 1:** Description of contours analyzed on each subject.

Contour Name	Description
Max Dose (MD)	The vessel contained in the PTV
Low-Dose Fed (LDF)	A vessel in the ipsilateral lung receiving between 5 and 20 Gy that branches downstream of the vessel irradiated to the max dose
No-Dose Fed (NDF)	A vessel in the ipsilateral lung receiving less than 5 Gy that branches downstream of the vessel irradiated to the max dose
Low-Dose Not-Fed (LDNF)	A vessel in the ipsilateral lung receiving between 5 and 20 Gy that does not branch from the vessel irradiated to the max dose
No-Dose Not-Fed (NDNF)	A vessel in the ipsilateral lung receiving less than 5 Gy that does not branch from the vessel irradiated to the max dose
Contralateral (CON)	A vessel in the contralateral lung (received no dose) at the approximate mirrored location as the point of max dose in the ipsilateral lung

**Table 2:** Percent changes in each measurement for each of the 6 contours analyzed. Each entry is the average percent change and standard deviation of the percent changes in each of the 5 (or 4 in the case of the no-dose fed contour) swine. Each entry is in the form “Average (Standard Deviation)”. Statistically significant values (significant at the alpha = 0.05 level) are noted with a * and in red.

	Max Rise	Max Value	Baseline to Baseline Time	Baseline to Baseline Difference	Slope Up	Slope Down	Area under Curve
**Max Dose** (Ipsilateral)	−40.7% (16.1%) *	−41.7% (18.1%) *	−26.3% (16.8%) *	−68.5% (23.1%) *	−43.0% (16.3%) *	−47.1% (19.2%) *	−56.0% (21.0%) *
**Low-Dose Fed** (Ipsilateral)	−42.6% (33.4%) *	13.6% (201.6%)	−3.9% (32.9%)	−125.0% (99.1%)	−47.7% (34.0%) *	−66.4% (25.9%) *	−65.0% (24.0%) *
**No-Dose Fed** (Ipsilateral)	−28.4% (47.8%)	−128.1% (232.6%)	−20.8% (32.0%)	−32.7% (9.7%) *	−50.6% (33.3%) *	−36.7% (42.4%) *	−55.0% (27.0%) *
**Low-Dose Not-Fed** (Ipsilateral)	−24.3% (52.6%)	−14.9% (68.8%)	−9.7% (43.9%)	−46.9% (41.0%)	80.3% (212.4%)	−38.6% (41.4%)	−36.0% (54.0%)
**No-Dose Not-Fed** (Ipsilateral)	−32.0% (32.0%)	−29.4% (35.3%)	3.1% (38.4%)	−47.7% (18.0%) *	79.9% (160.1%)	−1.3% (91.8%)	−24.0% (23%)
**Contralateral**	5.8% (15.2 %)	2.0% (12.1%)	−3.2% (33.2%)	−3.1% (5.0%)	40.5% (114.9%)	17.9% (40.1%)	−5.0% (25.0%)

## Data Availability

Not applicable.

## Supplementary Materials

The following are available online at https://www.mdpi.com/article/10.3390/jpm12081254/s1. Details regarding the use of wisconsin miniature swine.
