# Supplementary material for: Measuring Indirect Radiation-Induced Perfusion Change in Fed Vasculature Using Dynamic Contrast CT
Source: J Pers Med. 2022 Jul 30;12(8):1254. doi: 10.3390/jpm12081254 (PMC9410208; doi:10.3390/jpm12081254)
Supplement: Supplementary file 1 [file jpm-12-01254-s001.zip › jpm-1794539-supplementary.pdf]

SUPPLEMENTAL MATERIAL

DETAILS REGARDING THE USE OF WISCONSIN MINIATURE SWINE

Detailed Description of Methods

Indwelling Catheter Placement:

Dantrolene was administered prophylactically (5.9 mg/kg) in a small amount of feed to prevent against malignant hyperthermia. Anesthesia was administered using an injectable Telazol/Xylazine cocktail (3 mg/kg of Telazol and 1.5 mg/kg of Xylazine). Gas anesthesia was then administered via a nose cone (isoflurane at 2%). The subjects' necks were shaved and scrubbed bilaterally. Surgical depth of anesthesia was confirmed by testing toe pinch response and palpebral reflex. An indwelling central venous catheter was placed percutaneously in the vena cava (placement confirmed by fluoroscopy) and secured in place with suture. The catheter was sutured to the skin twice using finger-trap suture technique as well as using 2 butterfly clamps. The subject was outfitted with a pocketed catheter jacked, placed over a spandex shirt.

Prior to catheter placement 20 mL of blood was collected for serum and plasma samples. A 30 mL 0.9% saline flush was administered and anesthesia was discontinued. The subject was moved to the recovery pen for 2.5 hours before being returned to housing and given a feed ration.

Each imaging and fraction delivery session

Dantrolene was administered prophylactically (5.9 mg/kg) in a small amount of feed to prevent against malignant hyperthermia Anesthesia was administered using an injectable Telazol/Xylazine cocktail (3 mg/kg of Telazol and 1.5 mg/kg of Xylazine). Gas anesthesia was then administered via a nose cone (isoflurane at 2%). The subject was intubated and placed in a transport cart to be transported to the CT suite.

Once in the CT suite, Propofol was administered again (2.4 mg/kg) and Telazol (1.2 mg/kg) was administered in the left hind leg. The subject was mechanically ventilated at 15 breaths per minute and 1000 mL/breath. Mechanical ventilation of subject was periodically adjusted to appropriately maintain SPO2 and ETCO2 while allowing for desired image acquisition and irradiation. For all 4DCTs, ventilation was reduced to 8 breaths per minute and for all contrast enhanced CTs the subject was placed in an inspiration breath hold with a 1000 mL tidal volume. Two contrast-enhanced scans were performed. Each contrast scan involved injection of 80 mL of Omnipaque 300 followed by 50 mL of saline administered intravenously.

Once imaging was complete, ventilation, gas anesthesia, and saline administration were discontinued while the subject was transported from the CT suite to the LINAC vault. Once there, ventilation was resumed at 750 mL/breath and 8 breaths per minute with an inspiratory:

expiratory ratio of 2:1. Irradiation treatment of the lung was performed to deliver 12 Gy to the target.

Once irradiation was complete, anesthesia was discontinued and the subjects were weaned off mechanical ventilation. The subjects were transported to housing where they were extubated.

Description of the Dynamic Perfusion 4DCT

The dynamic 4DCT images were acquired over the central 15 cm of the lung as 80 ml of iodine contrast (Omnipaque 300) was injected at a rate of 5 ml/sec. The acquisition consisted of repeated scanning of the same volume at 1.5 sec intervals for 45 seconds and then every 4.5 seconds until the contrast had washed out of the lung.

The Dynamic CT given to the pigs is not given to humans as part of clinical workflow. We believe this scanning protocol is a better indication of perfusion than the standard blood volume dual energy scan because it scans the same volume over a period of time as contrast flows in and out of the vasculature as opposed to capturing a snapshot at one time point of where the contrast is in the lung.

Animal Care During Study

All WMS™ were housed on-site in facilities managed by the Biomedical Research Model Services group at the University of Wisconsin. Facilities undergo frequent inspection by the university Animal Care and Use Committee (ACUC) to ensure ethical treatment of animal subjects.
